# 

**Published:** 1872-07

**Authors:** 


### CONTENTS.

**COMMUNICATIONS.**

PAGE.

Hens and Health. Thos. K. Beecher....... 29
Wine & Brandy. P. H. Hayes, M. D....... 30
Things Sanitary. Ben. H. Pratt, A. M....... 32
Hernia. D. L. D. Sheldon, M. D....... 33
High Toned Quackery. Wm. R. McLean, M. D....... 34

**CORRESPONDENCE.**

Six Letters, and Answers to Correspondents....... 41

**EDITORIAL.**

PAGE.

Medical Items and News....... 35
Domestic Medicine....... 44
Abuses of the Eye....... 48
Watering Places....... 49
Sea-Sickness....... 50
Editorial Chit-Chat....... 51
Our Exchanges....... 55
New and Novel Machine....... 35
Innocent Amusement....... 35
Just Waked Up....... 44
Red Noses....... 56

